# Views on spousal support during delivery: a Turkey experience

**DOI:** 10.1186/s12884-018-1779-0

**Published:** 2018-05-09

**Authors:** Sermin Timur Taşhan, Yıldız Duru

**Affiliations:** 1 Nursing of Faculty. Department of Women Health and Diseases Nursing, Campus, Inonu University, Post Code: 44280 Malatya, Turkey; 2 Women Health Nurse, Elazığ Mental Health and Illness Hospital, Rızaiye Şehit Mehmet Güçlü Street, Post Code: 23200 Elazığ, Turkey

**Keywords:** Delivery, Women, women’s spouses, Spousal support, Social support

## Abstract

**Background:**

Spousal support is important during delivery since it satisfies the women and their spouses. Thus both women and their spouses should be included, where appropriate, in research on labour and birth. This descriptive study aimed to determine Turkish women’s and their spouses’ views on spousal support during delivery.

**Methods:**

The study population included women who stayed in the postpartum unit of a hospital and gave birth vaginally to their first child between the thirty-seventh and forty-second week of pregnancy. It also included their spouses. The study sample included 170 couples. The data were collected using introductory information forms administered to women and their spouses. The data were analyzed using percentages, averages, the chi-square test and logistic regression modeling.

**Results:**

This study indicated that 67.6% of the women and 71.8% of women’s spouses were in favor of spousal support during delivery. It also suggested that the women with negative experiences of childbirth needed social support during delivery and demanded to receive more spousal support but could not receive this support from the healthcare personnel (*p* < 0.05). The logistic regression models revealed that the risk of demanding to receive spousal support during delivery was 9.4 times higher in the women who needed social support during delivery than those who do not.

**Conclusion:**

This study demonstrated that women’s spouses wanted to be included in the delivery process more than the women wanted them to be, and the women who could not receive the necessary support needed more spousal support.

## Background

In many societies, childbirth is regarded as an act that is women’s responsibility. Opinions about childbirth are affected by economic and political changes, education, religious beliefs and social gender perception. In Turkey, childbirth is considered to be specific to women. Therefore, men’s involvement with it is limited in terms of health care approach and culture. This pushes men into a social situation that takes away their right to take part in the process of childbirth [1]. However, childbirth is a significant, special and exciting experience that a woman will have rarely during her lifetime. This experience takes women to a place of self-perception and integrity, their spouses to a place of trust and authority and the family to a place of power. However, it assigns important roles and responsibilities to both parents [1–3]. Pregnant women need support and feel that they need to be considered important in order to cope with delivery and have a healthy birth process. Professional support provided during the labor can provide women with the feelings of control and ability to cope with labor pain, and it can prevent negative experiences during delivery [4, 5]. Support can be provided by the spouse, relatives/family or a doula as well as midwives and nurses [6].

Men should accompany their spouses during delivery since this is important for the development of the family. There is an ever-increasing pressure for the men to accompany their spouses during delivery throughout the world [1, 2, 7]. With the increased number of prenatal training classes over the last two decades, men have begun to participate in delivery as well [1]. The presence of a woman’s spouse during delivery helps her cope with pain and prevents her from losing her control. Thus, spouses’ participation during delivery is considered to be beneficial and indispensable [7-9].

Although the rate of men’s participation in pregnancy, labor and postpartum processes has increased over the last three decades, the number of studies that were conducted in Turkey and examined men’s responsibilities during these times and the effect of fatherhood on men’s health is quite limited [1]. Studies conducted in Turkey indicated that the level of women’s needs for social support during delivery ranged between 70 and 82% [10, 11]. Spousal support during delivery varies in different countries and cultures, and this support can even differ within the same country [1, 3]. Studies on this issue included women’s views more while few studies examined their spouses’ views. Studies conducted in this field improve the skill for anticipating and satisfying the needs of healthcare personnel, women and their spouses as well as the efforts for providing family-centered care [12, 13]. Therefore, studies that will determine women’s and their spouses’ views on spousal support during delivery are necessary.

## Methods

### Sample and procedure

This descriptive study aimed to determine women’s and their spouses’ views on spousal support during delivery.

The study population included women who stayed in the postpartum unit of a hospital and their spouses. The study sample included 170 couples (340 people). The power analysis indicated that their effect size was 0.05, level of error was 5%, confidence interval was 95% and the level of representing the population was 98%. The sample was selected from the population using improbable accidental sampling method, and it included the women who stayed in the postpartum unit and their spouses. The specified hospital was the largest hospital with the highest number of childbirth. Moreover, it was the only place where postpartum mothers and their spouses could be contacted. Women and their spouses were included in the study until the sample size was achieved. Inclusion Criteria includes the following:

- Gave birth vaginally to the first child between the thirty-seventh and forty-second week of pregnancy.
- No development of a postpartum complication (obstructed labor, hemorrhage, toxemia, hematoma, infection, etc.) in women or infants.
- Having a single fetus.

Since the delivery experiences in the past could affect the current delivery experience, multiparous women were not included in this research.

Women who gave birth vaginally were discharged after being kept under observation for 6–24 h on average in the postpartum unit of the hospital. The hospital allowed the women to be visited only by female relatives and friends. Because of the cultural structure, male visitors including the spouses of the pregnant women were not permitted to attend the women while in hospital but almost all women had female companions. A total of fourteen midwives worked in the clinic. One physician and four midwives worked in the clinic during the day shift, and one physician and two or three midwives worked during the night shift.

The data were collected using introductory information forms which were delivered to women and their spouses and had been developed through previous studies [14–18]. These forms consisted of 13 questions for women and 8 questions for their spouses about their sociodemographic characteristics, opinions about the delivery experience and spousal support during delivery. The researcher aiming to collect data used face-to-face interview technique for five weekdays between September 12, 2011 and March 30, 2012. The data were collected first from the women and then from their spouses to prevent them affecting each other. The women completed the forms in approximately ten minutes while their spouses completed them in approximately five minutes.

Introductory Information Form for Women: This form included questions about the sociodemographic characteristics of the women (age, working status, educational status and income level), their delivery experiences (evaluation of the delivery, whether or not women saw their spouses and needed social support during delivery and other periods, and whether or not the required support was provided by the healthcare personnel) and their views on spousal support during delivery (demanding to receive spousal support during delivery, the reason for this demand, type of the demanded support, the period during which it was demanded and so on) [14–18]. Introductory Information Form for Men: This form included questions about the sociodemographic characteristics of the men (age, working status, educational status and income level) and the their views on spousal support during delivery (whether or not they were willing to provide support, the reason for being willing or reluctant, the type of support that was provided and so on) [14–18].

Before conducting the research, written permissions were obtained from the Elazığ Training and Research Hospital where the study was conducted and from the Biomedical Research Ethics Committee at the University of Inonu (No: 2011/113). The purpose and period of the study and the procedures to be performed were explained to the women and their spouses. They were told that they were free to withdraw any time they wanted. Written informed consent was obtained from all women and their spouses. Those who agreed to take part were included in the research.

The data were analyzed using the SPSS program for Windows 16.0 software package. The measurable data were presented in percentage (%), average (X) and standard deviation (SD). The data were analyzed using chi-square test and logistic regression modeling. The statistical significance threshold used was *p* < 0.05.

## Results

Table 1 presents the distribution of women and their spouses by their sociodemographic characteristics. Of the women, 71.2% were aged between 20 and 30 years, 82.4% did not work and 35.2% were secondary school graduates. Of the men, 67.6% were aged between 20 and 30 years, 82.4% worked and 58.2% were at least high school or university graduates. Moreover, of the women and their spouses, 54.7% reported that their family incomes were at the medium level. The research found no couples whose family incomes were sufficient (Table 1).

**Table 1:** Distribution of the sociodemographic characteristics of the women and their spouses (*N* = 340)

Sociodemographic Characteristics	Women		Women’s Spouses	
	*N*	%	*N*	%
Age^a^				
< 20	34	20.0	–	–
20–30	121	71.2	115	67.6
> 30	15	8.8	55	32.4
Working Status				
Yes	30	17.6	140	82.4
No	140	82.4	30	17.6
Educational Status				
Illiterate/Literate /Primary School	55	32.4	18	10.6
Secondary School	60	35.2	53	31.2
High School or University	55	32.4	99	58.2
Family Income				
Low	77	45.3	77	45.3
Medium	93	54.7	93	54.7
Total	170	100.0	170	100.0

^a^Mean age of the women: 23.5 ± 4.5 (Min: 16; Max: 37)

Mean age of women’s spouses: 28.6 ± 4.2 (Min: 21; Max: 43)

Table 2 indicates the distribution of the women’s delivery experiences and their views on spousal support during delivery.

**Table 2:** Distribution of women’s delivery experiences and their views on spousal support during delivery (*N* = 340)

Views on spousal support during delivery	Number	Percent
Delivery experience		
Positive	102	60.0
Negative	68	40.0
Need for social support		
Yes	109	64.1
Necessary support provided by the healthcare personnel		
Yes	123	72.4
The number of visits made by their spouses during their hospital stay		
Not seen	101	59.4
Once or twice	69	40.6
Demanding to receive spousal support during delivery		
Yes	115	67.6
Type of support expected from their spouses at the delivery (*n* = 115)^a^		
Emotional support	99	86.0
Physical support	16	14.0
Spousal support demanded (*n* = 115)^a^		
During the entire birth process	67	58.3
During the painful period of delivery	48	41.7
The reason for not demanding spousal support during delivery (*n* = 55)^b^		
No need	25	45.4
Embarrassment	23	41.9
Communication problems with the spouse	7	12.7

^a^replied by those who demanded to receive spousal support during delivery

^b^replied by those who did not demand to receive spousal support during delivery

Of the women, 60% considered their delivery experiences to be positive. Of them, 64.1% needed social support. This study found that 72.4% of the women were provided with the required support by the healthcare personnel. Of the women, 59.4% reported that they had not seen their spouses at all while staying at the hospital. Another finding that was not included in the table was that all women had female companions (Table 2).

In this study, 67.6% of the women wanted to receive spousal support during delivery. Of them, 86% wanted to receive emotional support from their spouses, which was the highest score in its category. 58.3% demanded to receive spousal support for the entire birth process, 45.4% reported that they did not want to receive spousal support because they did not need it, and 41.9% did not demand spousal support because they felt embarrassed (Table 2).

Table 3 indicates the distribution of the views of women’s spouses on spousal support during delivery.

**Table 3:** Distribution of the views of women’s spouses on spousal support during delivery (*N* = 340)

Views of women’s spouses on spousal support during delivery	Number	Percent
Willing to provide their spouses with support during delivery		
Yes	122	71.8
Type of the demanded support (*n* = 122)^a^		
Physical support (massage, helping women walk, breathing exercises etc.)	90	73.7
Emotional support	32	26.3
The period men wanted to participate in (*n* = 122)^a^		
The entire birth process	82	67.3
The painful period of delivery	40	32.7
The reason for being unwilling to participate in (*n* = 48)^b^		
The idea that there was no need for it	30	62.5
Fear	13	27.0
Embarrassment	5	10.5

^a^replied only by those who wanted to participate in the delivery

^b^replied only by those who did not want to participate in the delivery

Of the men, 71.8% reported that they were willing to provide their spouses with support during delivery. Of them, 67.3% stated that they wanted to be included in the entire birth process. A higher number of them (73.7%) wanted to provide physical support (massage, helping women walk, breathing exercises, etc.). Of the spouses who were unwilling to take part in the delivery, 62.5% stated that they did not want to participate because they thought there was no need for it (Table 3).

Table 4 indicates the distribution of women’s demands to receive spousal support by their social support experience.

**Table 4:** Distribution of women’s demands to receive spousal support by their delivery experience

Delivery Experience	Demanding to receive spousal support during delivery				Statistical Analysis*
	Yes		No		
	*N*	%	*N*	%	
Delivery experience					
Positive	60	58.8	42	41.2	X^2^ = 9.071
Negative	55	80.9	13	19.1	*p* = 0.003
Need for social support					
Yes	93	85.3	16	14.7	X^2^ = 43.357
No	22	36.1	39	63.9	*p* = 0.001
Necessary support provided by the healthcare personnel					
Yes	76	61.8	47	38.2	X^2^ = 6.977
No	39	83.0	8	17.0	*p* = 0.008

^*^X^2^: Chi square

Of the women who considered their delivery experience to be negative, 80.9% wanted to receive spousal support, and of the women who considered their delivery experience to be positive, 41.2% did not want to receive spousal support (*p* = 0.003). Of the women who needed social support, 85.3% reported that they did not want to receive spousal support. Of them who did not need, 63.9% stated that they did not demand to receive spousal support. Of the women who were not provided with the required support by the healthcare personnel, 83% wanted to receive spousal support, and of them who were provided with the required support by the healthcare personnel, 38.2% did not want to receive spousal support (*p* = 0.008) (Table 4).

The results of the logistic regression model are presented in Table 5. The results showed that women’s need for social support during delivery (OR = 9.40, 95% CI = 4.30–20.54) was a significant factor affecting the demand for spousal support during delivery.

**Table 5:** Risk factors of demanding to receive spousal support during delivery according to logistic regression model (*N* = 340)

Factors	β	SE^a^	df^b^	*p*	OR^c^	95% Cl^d^
Delivery experience (referent: Positive)						
Negative	.827	.47	1	.083	2.28	0.89–5.81
Need for social support (referent: No)						
Yes	2.241	.39	1	.000	9.40	4.30–20.54
Necessary support provided by the healthcare personnel (referent: Yes)						
No	.059	.56	1	.916	0.94	0.31–2.84

^a^*SE* Standard Error, ^b^*df* Degree of freedom, ^c^*OR* Odd’s ratio, ^d^*Cl* Confidence interval

## Discussion

Although social support provided during delivery is not an exact solution for decreasing women’s delivery stress, continuously providing them with emotional support and accompanying and encouraging them during delivery are some of the most effective ways of helping them feel safe and consider this process to be a positive one [3, 19, 20]. In the literature, the number of women considering their deliveries to be “easy and positive” ranged from 15.6 to 77.5% [11, 21–23]. The number of women who considered their delivery experiences to be positive shows similarity to those in the previous studies. However, the fact that a significant amount of women considering their delivery experiences to be negative should be scrutinized, and the reasons behind this fact should be examined.

More than half of the women reported that they needed social support, and this need was obvious during the entire birth process. The rate of women requiring social support during delivery was found to be as follows in previous studies: 83% in the study conducted by Turan et al. (2006); 75% in the study conducted by Morhason-Bello et al. (2008), and 74.5% in the study conducted by Timur and Hotun-Şahin (2010) [11, 16, 17]. The number of the relevant result of the present research was lower than those of the previous studies above. The difference is thought to be due to the hospital policies where the studies were conducted**.** In fact, female companions were allowed in the hospital where the present study was conducted. In this study, all women were accompanied by female companions, and this was probably why the rate of the required social support was lower than those of previous studies. Gender perceptions varying as a result of cultural differences may account for the difference in the findings of the studies.

The present study reported that three-quarters of women were provided with the required support by the healthcare personnel, and these women wanted to receive less spousal support. The previous studies showed that the level of support and care provided by the healthcare personnel ranged from 27 to 90% [11, 21, 23, 24]. The most important persons providing the support for the pregnant woman was the nurse and midwives, the only persons standing by the women and her family most often and establishing a close relationship with them [6]. The relevant finding of the present study was compliant with those of the previous studies. However, a limitation of the study was that the content of the support provided by the healthcare personnel was not questioned.

Over the last three decades, the rates of men’s participation in pregnancy, intrapartum and postpartum processes have increased [1]. In this study, more than half of the women wanted to receive spousal support during delivery. Udofia and Akwaowo (2012), Emelonye et al. (2017), Morhason-Bello et al. (2008) and Timur and Hotun-Şahin (2010) suggested that women wanted to receive support from their spouses mostly during delivery. The relevant finding of the present study showed similarity to the findings of similar previous studies [11, 16, 25, 26].

The present study demonstrated that the women wanted to receive emotional support from their spouses while the men planned to provide them with physical support. In the previous studies, women wanted to receive emotional and psychological support from their spouses with a level ranging from 50 to 86.6% [11, 16, 26, 27]. The relevant finding of the present study showed similarity to those of the previous studies. In the present study, the types of support that the women and their spouses favored were different from each other. This gives rise to the idea that spouses do not share their opinions on spousal support during delivery.

Although supportive interventions can be conducted both by spouses and nurses, the effect of the practices on women can vary according to whether they are performed by a beloved person or a person with knowledge and experience [28]. The previous studies showed that the support provided to women by the healthcare personnel or spouses had positive implications [3, 6, 27–30]. Women’s spouses reported that they wanted to participate in the delivery more than the women wanted them to participate. However, the unavailability of men during the childbirth is considered to be an important shortcoming that was overlooked in patient satisfaction and service quality. In fact, the study found that the women who considered their delivery experiences to be positive required less social support. The previous studies suggested that the support provided to women by the healthcare personnel or spouses had positive implications [3, 6, 9, 26–30]. The relevant finding of the present study was compliant with those of the previous studies.

The study found that most of women’s spouses wanted to take part in the entire birth process. However, the environmental pressure caused by hospital policies, cultural features and social gender perception makes it difficult for men to perform this action. This study’s husbands stated their reasons for being unwilling to participate in the delivery were “the idea that there was no need for it,” “fear” or “embarrassment,” which supports this idea. Gungor and Beji (2007) reported that 16% of them participated in the entire birth process while Ip (2009) stated that none of them took part in the entire delivery process [14, 27]. The research conducted by Gungor and Beji and the research conducted by Ip are experimental studies while this study is descriptive. Since the present study only examined men’s opinions, the extent of their availability during the childbirth is not actually known. The difference can be a result of the designs of the studies.

In some cultures, pregnancy and delivery are perceived to be “a woman’s job” [1, 3, 9]. The present study was conducted in the Eastern Turkey and in a region where patriarchal society characteristics were intensely observed. In relation to these cultural characteristics, this study found that the women did not want to receive spousal support because they were embarrassed or did not need it, while the main reason why women’s spouses did not want to provide spousal support was that they thought there was no need for it. Gungor and Beji (2007) reported that the reason why they did not want to be present in the delivery room at the moment of delivery was that their spouses (27.2%) did not want them to be there [14]. The relevant finding of the present study consistent with that of Gungor and Beji (2004) [14].

Although the number of the spouses was higher than that of the women, both were in favor of spousal support during delivery. Moreover, the women who considered their delivery experiences to be negative needed social support and could not receive this support from the healthcare personnel as they required more spousal support.

## Conclusions

Although the number of the spouses was higher than that of the women, both were in favor of spousal support during delivery. Moreover, the women who considered their delivery experiences to be negative needed social support and could not receive this support from the healthcare personnel as they required more spousal support.

## Abbreviations

Cld: Confidence interval
dfb: Degree of freedom
ORc: Odd’s ratio
SD: Standard deviation
SEa: Standard Error
X: Average
X^2^: Chi square
